# Preparation of Thifluzamide Polylactic Acid Glycolic Acid Copolymer Microspheres and Its Effect on the Growth of Cucumber Seedlings

**DOI:** 10.3390/ijms241210121

**Published:** 2023-06-14

**Authors:** Yuanyuan Li, Chaojie Wang, Xile Deng, Runze Cai, Lidong Cao, Chong Cao, Li Zheng, Pengyue Zhao, Qiliang Huang

**Affiliations:** 1 State Key Laboratory for Biology of Plant Diseases and Insect Pests, Institute of Plant Protection, Chinese Academy of Agricultural Sciences, Beijing 100193, Chinacaochong@caas.cn (C.C.); zhengli7seven@163.com (L.Z.); 2 Hunan Agricultural Biotechnology Research Institute, Hunan Academy of Agricultural Sciences, Changsha 410125, China

**Keywords:** polylactic acid-glycolic acid copolymer, thifluzamide, microspheres, fungicide delivery, cucumber growth

## Abstract

The polylactic acid-glycolic acid copolymer (PLGA) has been proven to be applicable in medicine, but there is limited research on its application and safety in the agricultural field. In this paper, thifluzamide PLGA microspheres were prepared via phacoemulsification and solvent volatilization, using the PLGA copolymer as the carrier and thifluzamide as the active component. It was found that the microspheres had good slow-release performance and fungicidal activity against *Rhizoctonia solani*. A comparative study was conducted to show the effect of thifluzamide PLGA microspheres on cucumber seedlings. Physiological and biochemical indexes of cucumber seedlings, including dry weight, root length, chlorophyll, protein, flavonoids, and total phenol content, indicated that the negative effect of thifluzamide on plant growth could be mitigated when it was wrapped in PLGA microspheres. This work explores the feasibility of PLGA as carriers in fungicide applications.

## 1. Introduction

At present, the control of agricultural diseases and insect pests mainly adopts the method of spraying large quantities of pesticides. In recent years, crop diseases have become increasingly serious. The use of fungicides has increased year by year, and the proportion in pesticide consumption is increasing gradually. However, excessive use of fungicides and unscientific application methods have led to problems, such as high production costs, excessive residue of agricultural products, and the development of disease resistance [1,2]. In the process of spraying, pesticide drift and liquid loss will occur, which not only affects environmental safety and economic interests but also causes too much toxicity and side effects of pesticide residues. It seriously violates the current policy of green environmental protection. Therefore, it is necessary to improve the effective utilization rate of fungicides [3,4,5,6,7].

It is found that the use of chemical materials loaded with fungicide to form a microsphere can effectively improve the utilization rate of fungicide, which has the advantages of long duration, high stability, low environmental pollution, and slow release [8,9,10]. Microspheres are a kind of physical controlled-release system. They mainly use biological multifunctional polymer compounds as the carriers, which can evenly wrap the active ingredients inside and be degradable in the environment. The active ingredients in the microsphere are gradually released through the process of dissolution, penetration, diffusion, and so on [11,12]. This avoids direct contact between the active ingredients and the external environment so that the active ingredients will not be affected by the changes in the external environment. Thus, the validity period of fungicide is prolonged, and some negative impacts of fungicide on the environment are reduced. Compared with other formulations, the controlled-release system has unique advantages, such as good targeting, slow release, high loading rate, and reduced toxicity. It has been applied to practice as a novel pesticide delivery system and extensively studied in the fields of medicine and biology [13,14]. For example, fungicide retention in encapsulation can reduce the reactivity with external factors, improve the physicochemical properties of active compounds, and enhance the bioavailability of active compounds [15,16]. Goetten de Lima et al. determined that a biocide delivery system composed of nanosilica loaded with neem oil is effective in reducing plant toxicity of this biocide [17]. In our previous study, we found that wheat seedlings treated with tebuconazole-loaded metal–organic frameworks could mitigate the negative effects of tebuconazole on the plant [18].

There are many materials used as carriers in microsphere preparation, such as starch, chitosan, polylactic acid, polylactic acid coordination agent, cellulose, and so on [19]. Among them, poly (lactic acid-glycolic acid) copolymer (PLGA) has good mechanical properties and low toxicity, and it has been widely used in the pharmaceutical industry [20,21,22,23,24]. It is made from the polymerization of two monomers, lactic acid and glycolic acid, with good biocompatibility and membrane-forming and cystic properties. PLGA is a non-toxic biodegradable polymer with excellent properties, which will not accumulate in the human body and will eventually be completely degraded into H_2_O and CO_2_. It was certified by the FDA and is officially listed in the United States Pharmacopoeia as a medicinal excipient [25]. As a carrier, it can extend the duration by slowly releasing the original active ingredients wrapped in it. Although PLGA is considered safe in the field of medicine, its safety as a pesticide carrier in agriculture has not been verified.

Cucumber is widely cultivated throughout the world. It is prone to lots of diseases during the planting period. In the seedling stage, cucumbers are susceptible to infection by *Rhizoctonia solani*, which leads to dead seedlings or mildew roots but also affects the contents of various endogenous hormones of cucumber, seriously reducing the quality and yield. The use of fungicides such as thifluzamide can effectively prevent and control this disease. Thifluzamide is an inhibitor of succinate dehydrogenase, which inhibits succinate dehydrogenase in tricarboxylic acid cycle of fungi and leads to fungal death. Thifluzamide is both preventive and curative and has strong internal absorption and conductivity and it can be applied in foliar spray, seed treatment, soil treatment, and other ways. Thifluzamide is also very effective against a wide range of fungal diseases and has special effects on sheath blight. It is reported that thifluzamide, as a seed treatment, can efficiently control major soil-borne diseases in the field [26]. It has obvious fungal inhibition on wheat sharp eyespot, take-all, and root rot of wheat through fungal inhibition tests [27]. As Thifluzamide has shown relatively excellent efficacy in the control of soil-borne diseases, such as peanut white sericea, Chinese herb standing blight, and vegetable root rot, it is accepted by more growers, and more compounding solutions and blending products are emerging, which have a broad market in the future. However, the improper use of thifluzamide can also have some negative effects on non-target organisms. It is shown that thifluzamide causes dysregulation of glucose metabolism and may lead to various thifluzamide-induced abnormal events in zebrafish [28]. Therefore, it is still necessary to consider the impact on the environment and non-target organisms, and it is very important to improve the safety of the use of agents.

PLGA has been widely used in the research and development of medical drugs, but there is relatively little research on pesticide formulations. In this study, thifluzamide microspheres were prepared using PLGA as the carrier. The poly (lactic acid-glycolic acid) copolymer no-load microspheres (P) and thifluzamide polylactic acid-glycolic acid copolymer microspheres (PT) were characterized, and the loading efficiency, release performance in vitro, and bioactivity of PT were also studied. The effects of P, PT, and a blank control (W) on the growth of cucumber seedlings were compared to investigate the safety of thifluzamide microspheres on the cucumber plant. This provides important support for the scientific and reasonable application of fungicide-loaded PLGA microspheres.

## 2. Results and Discussion

### 2.1. Optimization of Preparation Process

#### 2.1.1. Concentration of PVA

As seen in Table S1, under the premise that other preparation conditions were consistent, the loading rate and encapsulation rate increased with the concentration of PVA added for the first time The reason may be that the viscosity of PVA increases, which makes it difficult to touch the oil phase emulsion droplets, and the solidified microspheres have a better coating effect [29]. When the PVA concentration was 1% and 2%, there was no significant difference between the drug loading rate and encapsulation rate, and when the PVA concentration was 1%, the particle size of the microsphere was the smallest at 248.28 nm. When the concentration of PVA was 0.5% and 2%, the average particle sizes of the microspheres were 256.63 and 331.62 nm, respectively. Therefore, 1% was chosen as the optimal concentration for the first use of PVA.

#### 2.1.2. Concentration of PLGA

Table S2 shows that when the concentration of PLGA was 5 and 10 mg/mL, the encapsulation efficiency of PT was 21.15 and 19.37%, respectively. When the concentration of PLGA was 20 mg/mL, the encapsulation efficiency of PT was the highest, which was 46.07%. When the concentration of PLGA increased to a certain extent, the ratio of PLGA to thifluzamide would increase, which improved the encapsulation ability of PLGA to pesticides and reduced the possibility of a loss of active ingredients. Therefore, 20 mg/mL was selected as the optimal concentration of PLGA.

#### 2.1.3. Molecular Weight of PLGA

It can be seen from Table S3 that the molecular weight of PLGA has a certain effect on the particle size and drug loading rate of the microspheres. When the PLGA molecular weight ranged from 4000 to 15,000, the loading rate and encapsulation rate of the microspheres were 21.4%, 58.92%, and 248.28 nm, respectively. With the increase in molecular weight, the drug loading rate gradually increased, the particle size increased from 248.28 nm to 1347.75 nm, the degradation time became longer, and the release of active ingredients slowed down. In order to obtain smaller particle size and appropriate loading performance, PLGA with a molecular weight of 4000 to 15,000 was selected to prepare the microsphere.

#### 2.1.4. Oil–Water Ratio

DCM was used as the oil phase and PVA solution as the aqueous phase. The ratio of oil to water phase had a direct effect on the size of the microspheres. The distribution space of oil droplets would increase as the proportion of the water phase increased so that the average ultrasound energy that oil droplets received would reduce, and microspheres with large size would be obtained. As shown in Table S4, the oil–water ratio in the preparation process had an obvious effect on the loading rate, encapsulation rate, and particle size. After contrast optimization, the oil–water ratio was selected as 1:4, and the loading rate, encapsulation rate, and particle size of the microsphere were 21.4%, 58.92%, and 248.28 nm, respectively.

#### 2.1.5. Ultrasonic Power

As shown in Table S5, with the increase in ultrasonic power, the loading rate and encapsulation rate of the microsphere showed an increasing trend, but there was no significant difference between the loading rate and encapsulation rate under different power conditions. The ultrasonic power indicated the intensity of the input energy, and it had a significant effect on the particle size of the microsphere. The higher the ultrasonic power, the larger the particle size. Considering the particle size and encapsulation effect, 65 w was selected as the optimal ultrasonic power condition.

### 2.2. Characterization of PT and P

**Figure 1:**
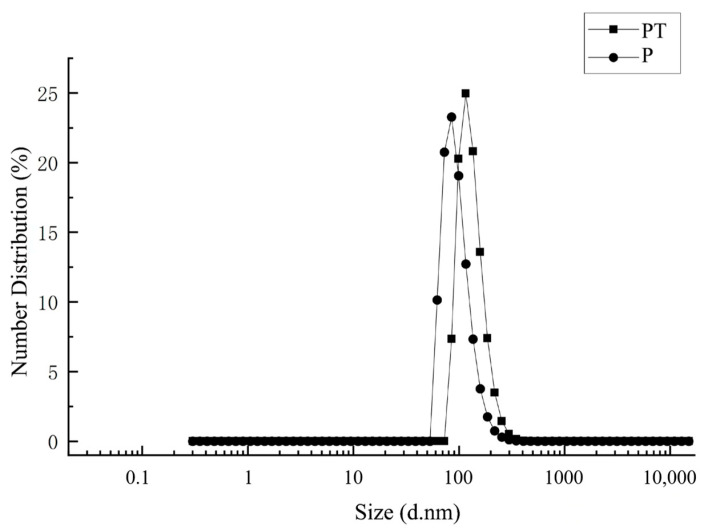
The particle size distribution of P and PT.

**Figure 2:**
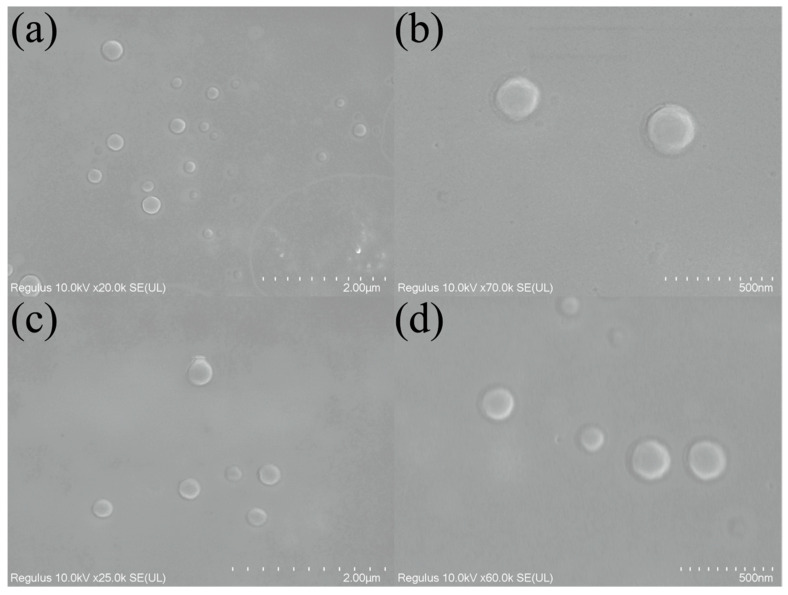
SEM images of PT (**a**,**b**) and P (**c**,**d**).

According to the measurement from the particle size analyzer, Figure 1 shows that the average particle size of P was 223 nm. After the fungicide was loaded, the average particle size of PT increased to 248 nm. The morphology of PT was observed via SEM. Both P and PT were uniform, round, and with a smooth surface. However, it can be observed from Figure 2 that the particle size of PT and P was about 200 nm, which was smaller than the data measured by the particle size analyzer. In the measurement of the particle size analyzer, the particles were suspended in water, and some water molecules bound to the surface of the particles, so the particle size may be larger than that observed under SEM. Due to the need to mix water in the actual use of fungicides, testing the particle size in suspension was more practical.

**Figure 3:**
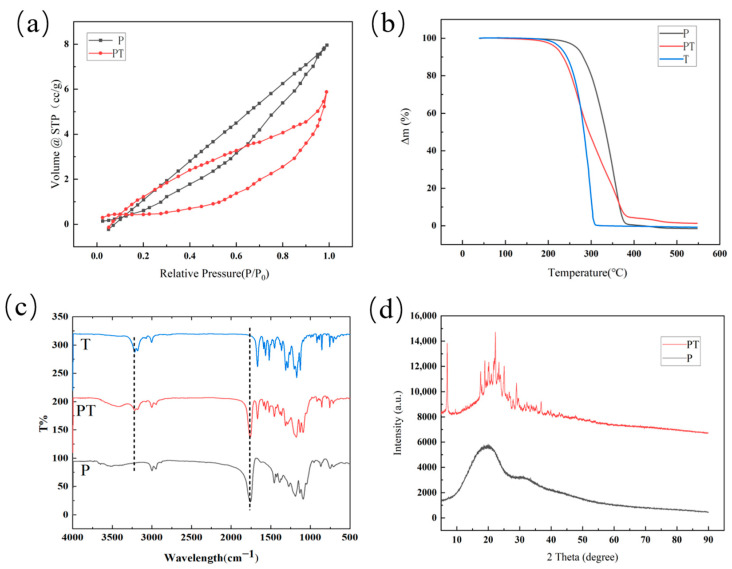
BET images of PT and P (**a**), TGA images of PT, P, and T (**b**), FT-IR images of PT, P, and T (**c**), XRD images of PT and P (**d**).

From the result of nitrogen adsorption/desorption measurement, there was an obvious decrease in nitrogen absorption after thifluzamide was loaded into PLGA, as shown in Figure 3a. Before loading thifluzamide, the BET surface area of the carrier was 9.312 m^2^/g, while that of PT decreased to 1.495 m^2^/g. After thifluzamide was coated by P, their surface area was reduced.

It can be observed from Figure 3b that from 200 to 300 °C, the weight of PT and thifluzamide (T) keeps a similar downward trend. In a temperature range of 200–300 °C, the weight of P decreases rapidly. Compared with the TGA curve of P, the TGA curve of PT was slightly different, which indicated that the thermal decomposition process of PLGA changed after thifluzamide was wrapped in PLGA.

FT-IR spectra of P and PT are shown in Figure 3c. T had a characteristic absorption at about 3200 cm^−1^, and this peak also appears in the IR diagram of PT, which proves that PT has successfully loaded the drug. The IR diagram of P showed characteristic absorption at around 1000–1500 cm^−1^ and 1700 cm^−1^, which also existed in the IR diagram of PT, indicating that the structure of PLGA did not change during the loading process [30,31,32].

The result of XRD analysis is shown in Figure 3d. There was a wide diffraction peak at 20° in P samples, which illustrated the amorphous structure in the PLGA polymer. As thifluzamide was a crystalline compound, there were many narrow and concentrated strong diffraction peaks in PT samples. The intensity of the diffraction peak in PT samples was higher than that in P samples when thifluzamide was coated in PLGA to form microspheres due to the interaction between thifluzamide and PLGA. The interaction inhibited the movement of thifluzamide molecules and, thus, reduced the crystallinity of thifluzamide in PT samples.

**Figure 4:**
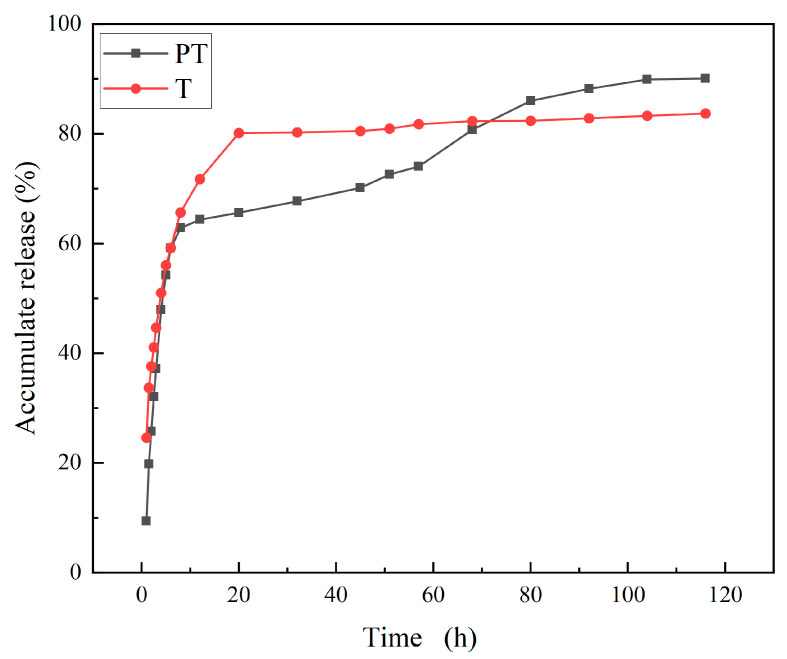
Release diagram of PT and T.

Figure 4 showed the release curves of PT and T in vitro. At the eighth hour, the release rates of PT and T were 62.82% and 65.65%, respectively. However, T continued to maintain rapid release, reaching 80% after 20 h and then stabilizing. On the other hand, the release rate of PT slowed down after 10 h, reached more than 80% after 80 h, and then remained stable. These findings suggested that PT had a sustained release effect. Furthermore, due to its small nanoscale size, PT had a solubilization effect, which improved the solubility of T in the release medium and resulted in a higher release rate.

### 2.3. Bioactivity

**Figure 5:**
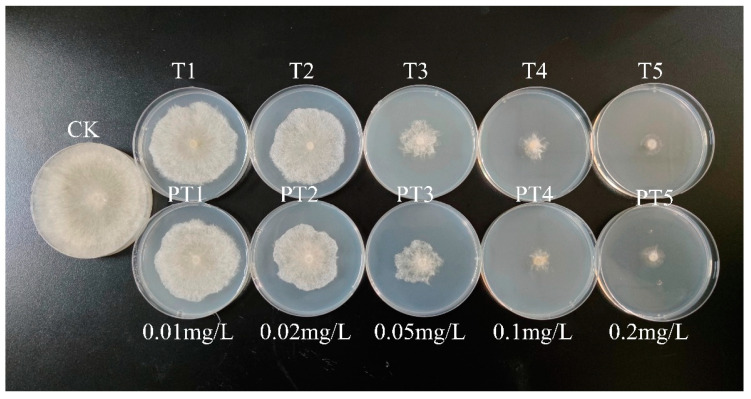
Determination of virulence of *Rhizoctonia solani*.

**Figure 6:**
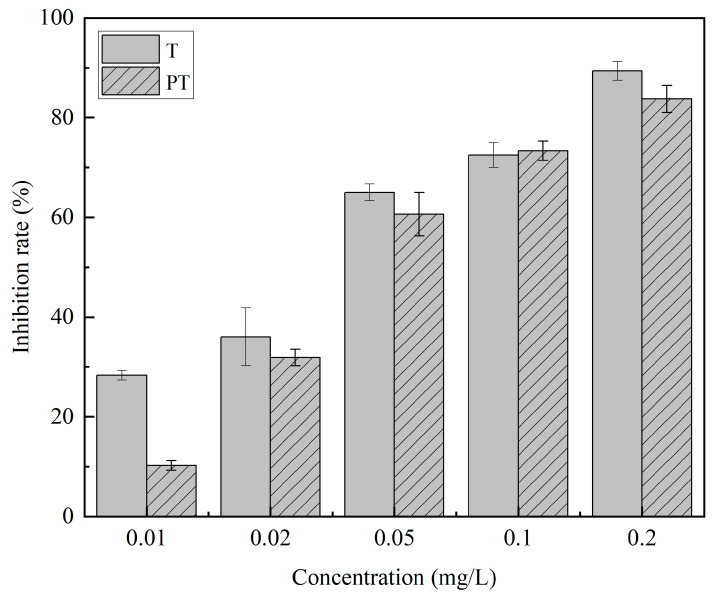
The inhibitory rates of the T and PT on *Rhizoctonia solani*.

The growth of *Rhizoctonia solani* treated with T and PT is shown in Figure 5. The bioactivity of active ingredients in PT was basically the same as that of free fungicides, which indicated that the loading process did not affect its bactericidal activity. The inhibitory rates of the T and PT on *Rhizoctonia solani* are shown in Figure 6. The inhibitory effect of T on *Rhizoctonia solani* was slightly higher than that of PT, and there was no significant difference in the concentration of 0.02–0.2 mg/L. The EC_50_ in the PT and T groups was 0.053 and 0.045 mg/L, respectively, and there was no significant difference in the PT and T groups. As a result, it indicated that the fungicidal activity was not changed during the fungicide loading process.

### 2.4. Effects on the Growth of Cucumber Seedlings

#### 2.4.1. Length and Weight

**Figure 7:**
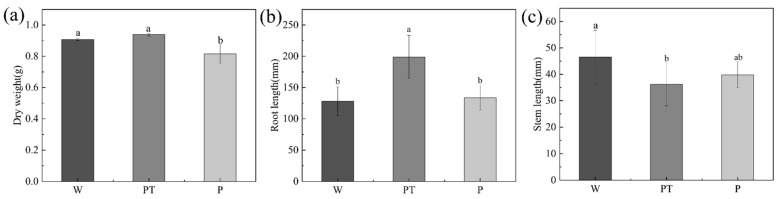
The average dry weight of cucumber plants after seven days of culture (**a**), the average root length of cucumber plants after seven days of culture (**b**), the average stem length of cucumber plants after seven days of culture (**c**). The different letters (a, b) on the columns represent the significantly different relationships between the results of the different treatments.

Parameters, such as root length, stem length, and dry weight, can be used as important indexes to evaluate the growth status of root cucumber seedlings. As can be seen from Figure 7, P decreased the dry weight, while PT did not differ significantly from the W group. The P and W groups had no significant difference in cucumber root length, while the PT group increased significantly. There was no significant difference between the P group and W group in cucumber stem length, while the PT group was significantly reduced compared with the W group.

#### 2.4.2. Chlorophyll

**Figure 8:**
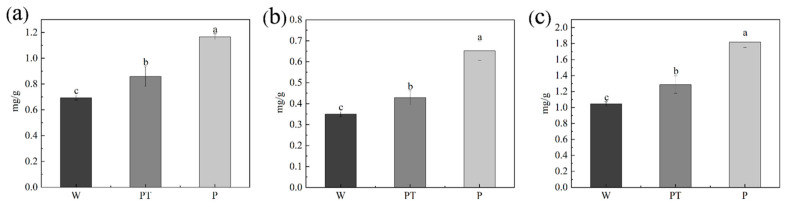
Chlorophyll A content in cucumber leaves (**a**), chlorophyll B content in cucumber leaves (**b**), total chlorophyll content in cucumber leaves (**c**). The different letters (a, b, c) on the columns represent the significantly different relationships between the results of the different treatments.

Photosynthetic pigments are not only the main substances for light energy absorption and transfer; essential electron transporters also play a role in the process of electron transfer. Plant chlorophyll is an important pigment involved in photosynthesis, so the smooth synthesis of chlorophyll is of great significance for the photosynthetic autotrophic ability of plants [33]. Some fungicides have been reported to be toxic to plants after application, affecting the light and structure of the leaves [34,35]. For example, triazole fungicides increase the non-photochemical quenching of cucumber plants [36]. Pyrazole and pyrimethanil can reduce the photosynthetic efficiency of wheat barley and soybean plants [37]. According to Figure 8, the contents of chlorophyll A, chlorophyll B, and total chlorophyll in the P group were the highest, reaching 1.167, 0.652, and 1.819 mg/g, respectively. Compared with other groups, the difference was significant. The chlorophyll A content in the W group was the lowest, which was only 0.694 mg/g, the chlorophyll B content in the W group was only 0.349 mg/g, and the total chlorophyll was also the lowest in the W group, which was 1.043 mg/g. The data showed that PLGA could promote the synthesis of chlorophyll in cucumber seedlings.

#### 2.4.3. Total Phenol, Total Flavonoids, and Protein

**Figure 9:**
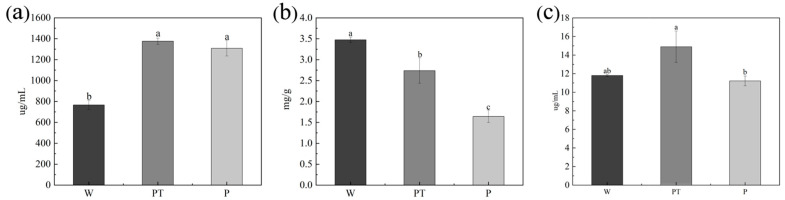
Protein content in cucumber leaves (**a**), total flavonoids content in cucumber leaves (**b**), total phenols content in cucumber leaves (**c**). The different letters (a, b, c) on the columns represent the significantly different relationships between the results of the different treatments.

Figure 9a shows the protein content of cucumber seedlings. The PT group had the highest protein content (1375 μg/mL), the P group was close to the PT group (1308.131 μg/mL), and the W group was significantly lower than the PT group (766.191 μg/mL). Figure 9b shows the flavonoid content of cucumber seedlings. As can be seen from the figure, there were significant differences among all groups, among which the flavonoids content of the W group was the highest (3.475 mg/g), and that of group P was the lowest (1.642 mg/g). Figure 9c shows the total phenol content of cucumber seedlings. The total phenol content in the PT group was the highest (14.904 μg/mL), and there was a significant difference between the P group (11.217 μg/mL) and PT group, while there was no significant difference between the W group (11.811 μg/mL) and PT group.

As many studies have shown, exogenous substances may change the content of phenols or flavonoids in plants, such as pesticide [38] or nanoparticles [39]. It was reported that phenols and flavonoids may inhibit seed germination and affect crop growth during the seedling stage [40]. In this work, it was found that P would decrease the total flavonoid content in cucumber seedlings and had no significant effect on the total phenol content. PT would increase the content of flavonoids and phenols, compared with the P group. Therefore, when thifluzamide was wrapped in PLGA polymers, its negative effect on plant growth could be mitigated.

## 3. Materials and Methods

### 3.1. Materials

Thifluzamide standard sample (95%) and technical material (95%) were obtained from National Center for Pesticide Quality Supervision and Inspection and Hellier Pharmaceutical Group Corporation. PLGA with molecular weight 37,000–52,000/10,000–20,000 was purchased from Shanghai Yuanye Biotechnology Co., LTD (Shanghai, China). PLGA with molecular weight of 4000–15,000 was purchased from Shanghai Maclin Biochemical Technology Co., LTD (Shanghai, China). Polyvinyl alcohol (PVA 1750 ± 50) was provided by Sinopharm Group Chemical Reagent Co., LTD (Ningbo, China). Dichloromethane (analytical grade) and methanol (chromatographic grade) were purchased from Tianjin Zhiyuan Chemical Reagent Co., LTD (Tianjin, China) and Tianjin Concord Technology Co., LTD (Tianjin, China), respectively. *Rhizoctonia solani* was isolated and purified from Plant Protection Institute of Chinese Academy of Agricultural Sciences. Tween-80 was purchased from Xilong Chemical Co., LTD (Shantou, China). The chlorophyll and protein kits were purchased from Jiancheng Bioengineering Institute, Nanjing, and the flavonoid kit and total phenols kits were purchased from Suzhou Grace Biotechnology Co., Ltd. (Suzhou, China).

### 3.2. Preparation of PT

Emulsification and solvent volatilization method is the most commonly used method to prepare microspheres. The principle of this method is to provide energy for the two kinds of originally incompatible liquids and make the molecules entangled and collide with each other to form emulsion. After the solvent of the internal dispersed phase is volatilized, the hardened material will automatically form microspheres [41,42]. This research adopts the emulsification–solvent evaporation method [43]. To prepare 20 mg/mL PLGA solution, 500 mg of PLGA was dissolved in 25 mL of methylene chloride (DCM). Then, 250 mg of thifluzamide was weighed and dissolved with DCM to prepare 10 mg/mL solution. After that, 2 mL of the thifluzamide solution and 2 mL of the PLGA solution were mixed evenly. While stirring, the mixed solution was added into 1% of PVA aqueous solution (16 mL) drop by drop. After stirring for 5 min, the mixed solution was treated using an ultrasonic mixer (Ymnl-650Y, Immanuel Instrument Co., LTD, Honolulu, HI, USA) for 2 min. The obtained emulsion was added into 32 mL of 0.05%PVA solution, which was stably dispersed for 20 min, and then DCM was evaporated through rotating evaporation for 10 min. The mixture was separated via centrifugation at 10,000 r/min for 10 min. The precipitates were washed with water 3 times. Finally, they were freeze-dried to obtain the fungicide microsphere.

### 3.3. Characterization

The obtained samples of P and PT were characterized using scanning electron microscopy (SEM), Fourier-transform infrared (FT-IR), thermogravimetric analysis (TGA), and Brunauer–Emmett–Teller (BET) surface area. The loading efficiency, release performance in vitro, and bioactivity of PT were also studied.

The particle size of the microsphere was measured using a laser particle size distributor (BT-9300 H, Dandong Baxter Instrument Co., LTD, Dandong, China). The quantity average particle size was selected as the particle size of the microsphere in this experiment. The particle size was continuously measured 3 times using the laser particle size distributor. The microsphere morphology was observed via SEM (Hitachi S-4800, Hitachi Ltd., Tokyo, Japan). The acceleration voltage was set at 10 kV. PT water solution was dropped on the conductive tape and then dried at room temperature. The samples were coated with gold before imaging.

TGA was performed on a NETZSCH TG 209 F3 thermal system (Wood-land, German) from 20 °C to 550 °C at a rate of 10 °C/min. Then, 4 mg of samples was loaded into an alumina crucible for testing. A nitrogen atmosphere was employed. The nitrogen adsorption/desorption isotherms of P and PT were then measured using a TriStar II 3020 (Quantachrome Instruments Quadrasorb EVO, Beach, FL, USA). The prepared material was dried prior to gas adsorption measurements. Samples larger than 100 mg were weighed and vacuumed at 15 °C. Nitrogen was used as analysis gas and degassed for 12 h to determine saturation pressure. BET equation was used to calculate specific surface area.

FT-IR was measured using an infrared spectrometer (Thermo Scientific Nicolet iS5, Waltham, MA, USA). The potassium bromide press method was used with a measured transmission range of 400–4000 cm^−1^, a scan time of 64, and a resolution of 4 cm^−1^. XRD measurements were performed on an X-ray diffractometer (D8 Advance, Germany Brock). Test conditions were voltage 40 KV, current 40 mA, step size 0.02°, test speed 0.1 s/step, copper target, and incoming ray wavelength 0.15406 nm.

The loading rate of PT was determined using HPLC (DIONEX UltiMate3000, Thermo Scientific, Waltham, MA, USA). Further, 10.0 mg of PT samples was weighed and then 15 mL of methanol was added to dissolve them via ultrasound for 3 h. After that, 1 mL of the suspension was extracted and filtered with 0.22 μm filter membrane for HPLC injection. The mobile phase of HPLC was methanol/water (75/25, *v*/*v*). A ZORBAX SB-C18 column (Agilent, 250 mm × 4.6 mm, 5 μm) was used. The sample volume was set as 5 μL, and the chromatographic column temperature was kept at 30 °C, with a mobile phase flow rate of 1.0 mL/min. The detection wavelength was 230 nm. The average of the 3 determinations was taken.

Controlled-release performance of PT in vitro was determined. Thus, 80% methanol aqueous solution was prepared as the release medium. Before controlled-release test, the dialysis bag with the interception molecular weight of 3500 was cut into 8 cm long and soaked in pure water for 24 h. Then, 30 mg of PT was accurately weighed into the dialysis bag, and 3 mL of release medium was added. Then, the dialysis bag was placed in a release bottle containing 197 mL of release medium. The mixture was shaken in a thermostatic oscillator (HZQ-X300C, Shanghai Yiheng Scientific Instrument Co., LTD, Shanghai, China) at 150 rpm. At intervals, 0.7 mL of solution was extracted for HPLC analysis. The same volume of fresh release medium was added to keep the total volume. The cumulative release was calculated as in our previous study [44].

### 3.4. Bioactivity Test of PT

In order to determine the biological activity of PT, the bactericidal activity of PT was determined from the mycelium growth rate method with *Rhizoctonia solani* as the test strain. The sterile molten potato dextrose agar (PDA) containing 0.2% of Tween-80 was used to mix with PT and T, respectively. A mycelial disc with a diameter of 5 mm was inoculated on the PDA plates. In both treated and control groups, the concentration gradient of active ingredient was set as 1, 2, 5, 10, and 20 mg/L. The incubation temperature was set at 28 °C and the relative humidity was kept at 85%. After 3 days, the growth diameter of each colony was measured, and the growth inhibition rate (%) was calculated.

### 3.5. Cucumber Culture and Treatment

Healthy and plump cucumber seeds were selected and disinfected with 0.3% sodium hypochlorite for 15 min. After being rinsed with ultrapure water 3–4 times, they were soaked in warm water at 55 °C for 10 min and then kept in water for 4 h at room temperature. The seeds were planted in a seedling tray, and the culture conditions were day/night (12 h/12 h), 33/25 °C, and 60% humidity. In the trilobal period, the cucumber seedlings were cultured in P and PT solutions. The concentrations in both treated groups were set as 50 and 250 mg/L with 0.5% Tween-80. The concentration was determined with reference to the recommended dose and the results of pre-tests, which showed that this concentration was effective in suppressing the disease without causing harm. Blank comparison (W) group was set during the treatment. The cucumber seedlings were cultivated for 7 days, and they were collected for physiological and biochemical determination.

### 3.6. Determination of Physiological and Biochemical Indexes of PT

Fresh cucumber seedlings were heated in an oven at 105 °C for 30 min, then kept at 80 °C until the weight remained unchanged, and the dry weight was recorded. Other fresh seedlings were ground into powder in liquid nitrogen with mortar and pestle and stored at −80 °C. Chlorophyll A, chlorophyll B, total chlorophyll, protein, total flavonoids, and total phenols were measured using the corresponding detection kit. We then extracted and determined samples according to the instructions. The content of protein was determined using bicinchoninic acid (BCA) microplate. The total flavonoids were determined via the color development method of NaNO_2_-Al (NO_3_)_3_-NaOH, and the total phenols were determined through the method of flint phenol. The absorbance of different treatments was measured using Clariostar microplate reader (BMG Labtech, Cary, NC, USA).

## 4. Conclusions

PLGA microspheres can extend the duration by slowly releasing the original active ingredients wrapped. They are considered safe in the field of medicine, but the safety as pesticide carriers in agriculture has not been reported. In this work, thifluzamide PLGA microspheres were successfully prepared with a nanometer range and good controlled-release performance. There was no significant difference in fungicidal activity between the thifluzamide microsphere-free thifluzamide. The different effects of PLGA and thifluzamide microspheres on cucumber seedlings were studied using a comparative experiment, such as dry weight, root length, chlorophyll, protein, flavonoids, and total phenol content. As carriers, PLGA could reduce the toxicity of free thifluzamide to cucumber seedlings. This work provided some new information for the application of PLGA fungicide microspheres.

## Data Availability

Research data are available upon request to the authors.

## Supplementary Materials

The supporting information can be downloaded at: https://www.mdpi.com/article/10.3390/ijms241210121/s1.

## Abbreviations

PLGA	polylactic acid-glycolic acid copolymer
PT	thifluzamide polylactic acid-glycolic acid copolymer microspheres
P	poly (lactic acid-glycolic acid) copolymer no-load microspheres
W	blank comparison
T	thifluzamide
PVA	polyvinyl alcohol
DCM	methylene chloride
PDA	potato dextrose agar
BCA	bicinchoninic acid
SEM	scanning electron microscopy
FT-IR	Fourier-transform infrared
TGA	thermogravimetric analysis
XRD	X-ray diffraction
BET	Brunauer–Emmett–Teller
HPLC	High-performance liquid chromatography
