# Supplementary material for: Preparation of Thifluzamide Polylactic Acid Glycolic Acid Copolymer Microspheres and Its Effect on the Growth of Cucumber Seedlings
Source: Int J Mol Sci. 2023 Jun 14;24(12):10121. doi: 10.3390/ijms241210121 (PMC10299088; doi:10.3390/ijms241210121)
Supplement: Supplementary file 1 [file ijms-24-10121-s001.zip › ijms-2401926-supplementary.pdf]

Preparation of Thifluzamide Polylactic Acid Glycolic Acid Copolymer Microspheres and Its Effect on the Growth of Cucumber Seedlings

Yuanyuan Li[1], Chaojie Wang[1], Xile Deng[2], Runze Cai[1], Lidong Cao[1], Chong Cao[1], Li Zheng[1], Pengyue Zhao[1*], Qiliang Huang[1*]

[1] State Key Laboratory for Biology of Plant Diseases and Insect Pests, Institute of Plant Protection, Chinese Academy of Agricultural Sciences, Beijing 100193, China

[2] Hunan Agricultural Biotechnology Research Institute, Hunan Academy of Agricultural Sciences, Changsha 410125, Hunan, China

[*] Correspondence: zhaopengyue@caas.cn (P. Zhao); qlhuang@ippcaas.cn (Q. Huang)

Table S1. Effects of different PVA concentrations on quality technical indexes of PT

PVA concentration (%)	Loading rate (%)	Encapsulation rate (%)	Particle size (nm)
0.5	16.29	43.17	256.63±1.28
1	21.4	58.92	248.28±1.08
2	22.5	60.73	331.62±7.00

Table S2. Effects of PLGA concentrations on quality technical indexes of PT

PLGA concentration (mg/mL)	Loading rate (%)	Encapsulation rate (%)	Particle size (nm)
5	32.54	21.15	239.42±1.29
10	27.67	19.37	403.44±8.23
20	22.47	46.07	248.28±1.08

Table S3. Effects of PLGA molecular weight on quality technical indexes of PT

PLGA molecular weight (Da)	Loading rate (%)	Encapsulation rate (%)	Particle size (nm)

4000-15000	21.4	58.92	248.28±1.08
10000-20000	22.47	60.93	405.46±5.33
30000-40000	28.69	74.59	1347.75±475.43

Table S4. Effects of oil-water ratio on quality technical index of PT

Oil-water volume ratio (V/V)	Loading rate (%)	Encapsulation rate (%)	Particle size (nm)
1:2	20.95	62.85	278.49±2.50
1:4	21.4	58.92	248.28±1.08
1:8	26.96	62.01	421.19±5.20

Table S5. Effects of ultrasonic power on quality technical index of PT

Ultrasonic power (w)	Loading rate (%)	Encapsulation rate (%)	Particle size (nm)
65	21.4	58.92	248.28±1.08
104	22.53	60.85	264.33±0.47
130	24.08	61.79	279.02±5.04

The loading rate and encapsulation rate of thifluzamide PLGA microspheres were calculated according to the following equation:

Loading rate (%) = $\frac{m_1}{m_2}$,

Encapsulation rate (%) = $\frac{m_3}{m_4}$,

m_1 is the mass of thifluzamide in the sample,

m_2 is the total mass of the sample,

m_3 is the actual loaded mass in the sample,

m_4 is the theoretical loaded mass in the sample.
